# Ipvelutine, 7β-Acetoxy-2α-(tigloyloxy)tropane, an Unusual Tropane Alkaloid from *Ipomoea velutina* R. Br. (Convolvulaceae)

**DOI:** 10.3797/scipharm.1303-13

**Published:** 2013-06-04

**Authors:** Sonja Christina Ott, Kristina Jenett-Siems, Karsten Siems, Frank Müller, Monika Hilker, Eckart Eich

**Affiliations:** 1 Institut für Pharmazie (Pharmazeutische Biologie), Freie Universität Berlin, Königin-Luise-Str. 2-4, D-14195 Berlin, Germany.; 2 AnalytiCon Discovery, Hermannswerder Haus 17, D-14473 Potsdam, Germany.; 3 Institut für Biologie (Angewandte Zoologie/Ökologie der Tiere), Freie Universität Berlin, Haderslebener-Str. 9, D-12163 Berlin, Germany.

**Keywords:** *Ipomoea velutina*, Convolvulaceae, Ipvelutine, 7β-Acetoxy-2α-tigloyloxytropane, 2,7-Disubstituted Tropanes, Structure Elucidation

## Abstract

Convolvulaceae provide a rich source of tropane alkaloids, however, 2-substituted tropanes have been described for only few species of this taxon. In this note, 2,7-diesters such as ipvelutine [7β-acetoxy-2α-(tigloyloxy)tropane] isolated from the vegetative parts of the Australian *Ipomoea velutina* R. BR. are described as a new group of tropane diesters.

## Introduction

During our continuous studies on secondary metabolites of the Convolvulaceae, this plant family has been shown to produce a plethora of tropane alkaloids, especially 3-tropanols and their esters (e. g. [1, 2]), as well as some 3,6-disubstituted tropanes [3] or the polyhydroxylated calystegines [4]. This underlines the chemotaxonomic relationship with their sister family Solanaceae where the biosynthetic pathway of tropane alkaloids is well investigated. The main route leads to two stereoisomeric 3-hydroxytropanes, namely 3α-tropanol (basic component of the well-known atropine and other esters), and 3β-tropanol which is also precursor of the calystegines. 2-Substituted tropane alkaloids could only be found as a by-product in the Solanaceae [5]. Accordingly, amongst the tropane alkaloids of the Convolvulaceae 2-substituted ones are extremely rare, too, and could only be detected in some *Calystegia*, *Erycibe*, and *Ipomoea* species [6].

## Results and Discussion

In the alkaloidal screening of Convolvulaceae via GC-MS analysis the basic extracts of the Australian *Ipomoea velutina* R. Br. revealed the presence of several unknown substances. In the basic extract of the vegetative parts seven unknown nitrogen-containing compounds were detected: one main alkaloid and six minor ones (0.7–18.7% of the main alkaloid by integration of the corresponding GC-MS peaks). The molecular formula of the main compound (**1**) is consistent with C_15_H_23_NO_4_ (*m/z* 281).

The ^1^H-NMR (Table 1) in combination with HSQC and HMBC experiments showed two acylic residues: a C_5_-acid containing a double bond, namely tiglic acid, as well as acetic acid. Both were confirmed by fragmentation ions in the EIMS as products of α-cleavage neighbouring the ester carbonyls: *m/z* 83 (C_4_H_7_−CO^+^; HRMS: [C_5_H_7_O]^+^ as 83.04959, calcd. 83.04969) and *m/z* 43 (CH_3_−CO^+^).

**Fig. 1:**
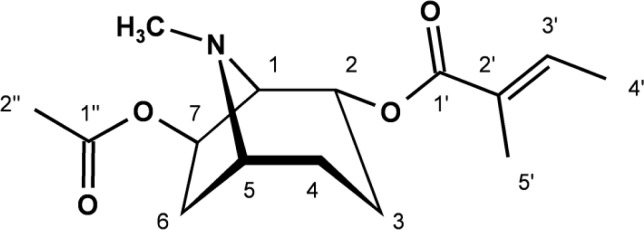
Structure of ipvelutine [7β-acetoxy-2α-(tigloyloxy)tropane], main alkaloid from the vegetative parts of *Ipomoea velutina* R. Br.

The HSQC spectrum revealed a characteristically downfield shifted N−CH_3_ (δ_C_ 40.9, δ_H_ 2.91) as well as three methylene signals (δ_C_ 37.8, 27.5, and 22.8) and four methine groups (δ_C_ 72.9, 70.8, 68.9, and 64.7). From the ^1^H-^1^H-COSY, the complete coupling sequence could be deduced. As a result, **1** (Fig. 1) could be identified as a 2,7-disubstituted tropane.

The substitution pattern of the tropane diester was derived from the mass spectrometric data on the basis of the specific mass fragmentation in bridge-substituted tropanes. The most important fragment is [M − X−COO−CH=CH_2_]^+^ after expulsion of the ethylene bridge C-6−C-7 including its substituent; this allows a prediction of the substituents’ positions in 3,6/7-disubstituted tropanes [7, 8]. Regarding **1**, there are two possible key ions: in case of acetylation in position 7 *m/z* 195 or in case of acetylation in position 2 *m/z* 155. As there is only a veritable peak at *m/z* 195, **1** has to be acetylated in position 7 of the tropane.

The relative stereochemistry of **1** was deduced from characteristic coupling constants: H-7 showed a doublet-doublet with coupling constants of 3.4 Hz and 7.9 Hz that can also be observed in the 7β-substituted schizanthines C–E [9]. This corresponds with the experience that, for steric reasons, bridge substituents usually are *exo*-orientated. H-2 showed a *trans*-diaxial coupling constant *J* = 10 Hz which is – according to [10] and [11] – specific for α-orientated substituents at C-2. These conclusions were also confirmed by NOE measurements: H-2 (δ_H_ 5.02) showed correlations to H-1 (δ_H_ 3.55), to the equatorial H-3e (δ_H_ 1.98) and to the axial H-4a (δ_H_ 1.89) which is only possible if H-4a and H-2 are both axial [11]. H-7 (δ_H_ 4.61) was correlated to H-1 (δ_H_ 3.55) and – only enabled by its *endo*-position – to the axial H-3a (δ_H_ 1.49) and H-6n (δ_H_ 2.36).

Thus, **1** (ipvelutine) was identified as 7β-acetoxy-2α-(tigloyloxy)tropane.

In the vegetative parts and/or roots, eight minor compounds related to ipvelutine could be detected by GC-MS analysis. They were identified by their fragmentation patterns; characteristic base peaks of those 2,7-disubstituted tropanes are *m/z* 95 and *m/z* 82 or *m/z* 81 together with a prominent peak at *m/z* 156, and of their *nor*tropane derivatives *m/z* 125 and *m/z* 81 including a half-maximal peak at *m/z* 108. An additional result of the systematic GC-MS screening is the detection of ipvelutine (appearing as deacetylated derivative in GC-MS analysis) in vegetative parts of *Convolvulus graminetinus*, *C. sagitattus*, and *Ipomoea abrupta*. Both *Convolvulus* species afforded similar structures, as well, and, additionally, the corresponding *nor*tropanes in the roots. Ipvelutine-related substances were also found in *Ipomoea asarifolia* and *I. plebeia*. The mass fragmentation patterns obtained by GC-MS analysis show that these variations include differences in the stereostructure at C-2 or/and C-7, alternation of the position of the substituents, methylbutyric and hydroxymethylbutyric acid as diverging acyl components, change of the bridge substituents’ position from C-7 to C-6 and a hydroxy group as additional substituent (for details see [12]).

2,7-Dihydroxy*nor*tropane showing the same substitution pattern as ipvelutine is also synthesized by root cultures of *Calystegia sepium* (Solanaceae). Incorporation experiments with ^15^N-labelled 3-tropanone revealed that, unless 2,7-dihydroxy*nor*tropane derives the regular tropane alkaloid pathway, it is not an intermediate in calystegine biosynthesis, but can be seen as a by-product [5].

From the pharmacological point of view, the finding of ipvelutine and derivatives is of interest since they show structural similarity to bao gong teng A [13] obtained from the vegetative parts of *Erycibe obtusifolia* (Convolvulaceae). Bao gong teng A is characterized by strong miotic properties and therefore used as an antiglaucoma agent in medicinal products. This pharmacological effect is contradictory to that of atropine/hyoscyamine having significance as a mydriatic in ophthalmology and being one of the most commonly used tropanes of natural origin.

## Experimental

### General procedures

^1^H-NMR and ^1^H-^1^H-COSY spectra were obtained on a Bruker AMX 400 MHz, HSQC and HMBC spectra on a Bruker DRX 500 MHz (TMS as internal standard). EIMS and HR-EIMS were recorded on a Varian MAT 711 (80 eV), FABMS on a Varian MAT CH_5_DF. The GC-MS system consisted of a Fisons GC 8060 coupled to a quadrupole mass spectrometer Fisons MD 800c.

### Plant material

Roots and vegetative parts of *Ipomoea velutina* R. Br. grown from seeds collected in the wild at Florence Falls, Litchfield National Park, Northern Territory/Australia, were harvested in the greenhouse of the Institut für Pharmazie, Freie Universität Berlin. A voucher specimen is deposited at the herbarium of the Berlin-Dahlem Botanical Garden – Botanical Museum (BGBM), Freie Universität Berlin, Germany.

### Extraction and isolation of ipvelutine

235 g dried and ground vegetative parts of *Ipomoea velutina* were extracted 4 h with 3 L MeOH three times and once with a mixture of 2.4 L MeOH and 600 mL 2% aqueous tartaric acid. After evaporation of the MeOH (50°C i. V.), the residue was redissolved in 600 mL 2% aqueous tartaric acid and extracted with petrol ether, CH_2_Cl_2_, and EtOAc, respectively (3 × 500 mL each). Then, the aqueous layer was alkalinized (pH 10) with aqueous NH_3_ (25%) and extracted with 4 × 500 mL CH_2_Cl_2_. The united alkaline CH_2_Cl_2_ fractions gave 172 mg crude alkaloid fraction which was dissolved in 50 mL 2% aqueous tartaric acid again and extracted with petrol ether, CH_2_Cl_2_, and EtOAc (3 × 50 mL each). After addition of aqueous NH_3_ (pH 10), the aqueous layer was extracted with 4 × 50 mL CH_2_Cl_2_. After drying over Na_2_SO_4_ and evaporation of CH_2_Cl_2_ (40°C i. V.), the alkaline fractions were united and 10 mg ipvelutine were gained (81% purity according to NMR spectra).

#### 7β-Acetoxy-2α-(tigloyloxy)tropane [(1S,2S,5R,7R)-7-(acetyloxy)-8-methyl-8-aza-bicyclo[3.2.1]oct-2-yl (2E)-2-methylbut-2-enoate, ipvelutine, **1**]

Yellow oil. ^1^H-NMR (400 MHz, MeOD): see Table 1. ^13^C-NMR (100.6 MHz, MeOD): see Table 1. MS (EI, 80 eV, 110°C): *m/z* (%) = 281 (2) [M]^+^, 239 (83), 195 (7), 156 (100), 142 (60), 140 (35), 112 (11), 98 (46), 96 (84), 95 (91), 94 (50), 85 (41), 84 (31), 83 (27), 55 (22), 43 (20). (+)-FAB MS (80 eV): *m/z* = 282 [M+H]^+^. HR MS (80 eV): *m/z* = 281.16256 (calcd. 281.16271 for C_15_H_23_NO_4_), 239.15283 (calcd. 239.15214 for C_13_H_21_NO_3_), 156.10254 (calcd. 156.10245 for C_8_H_14_NO_2_^+^), 142.08678 (calcd. 142.08681 for C_7_H_12_NO_2_^+^), 140.10749 (calcd. 140.10754 for C_8_H_14_NO^+^), 98.062524 (calcd. 98.06059 for C_5_H_8_NO^+^), 95.072728 (calcd. 95.073499 for C_6_H_9_N).

### GC-MS analysis

Ground plant parts (50 g) were extracted three times with 500 mL MeOH (80%). After evaporation the residue was dissolved in 2% aqueous tartaric acid and extracted with petrol ether, CH_2_Cl_2_, and EtOAc. The aqueous layer was alkalinized and extracted with CH_2_Cl_2_. To purify the extracts obtained, this procedure was repeated with corresponding smaller amounts of the solvents. The resulting extracts were subjected to GC-MS analysis. Samples were injected at 240°C (split 1:20) and separated on a DB-1 column (0.32 mm ×30 m, J&W Scientific, California) by raising temperature from 70°C to 300°C at 6°C/min. Helium was used as carrier gas. Retention indices (RI): Kovats indices [14] were calculated in relation to a set of co-injected hydrocarbons.

## Figures and Tables

**Tab. 1:** ^1^H- and ^13^C-NMR data of ipvelutine (in MeOD)

**atom**		**^1^H-NMR (in MeOD)**		^13^C-NMR* (in MeOD)
1	3.55	*br d*	3.2 Hz	72.9
2a	5.02	*ddd*	2.2 Hz; 5.9 Hz; 11.3 Hz	68.9
3e	1.98	*m*		22.8
3a	1.49	*dtd*	6.4 Hz; 12.1 Hz; 12.8 Hz	
4a	1.89	*m*		27.5
4e	1.68	*ddd*	2.3 Hz; 6.7 Hz; 13.7 Hz	
5	3.82	*br t*	5.2 Hz	64.7
6n	2.36	*dd*	8.0 Hz; 14.6 Hz	37.8
6x	2.27	*ddd*	3.5 Hz; 6.3 Hz; 14.7 Hz	
7n	4.61	*dd*	3.4 Hz; 7.9 Hz	70.8
N−CH_3_	2.91	*s*		40.9
1’				167.8
2’				129.0
3’	6.96	*dq*	1.2 Hz; 6.9 Hz	139.3
CH_3_−4’	1.83	*d*	7.1 Hz	11.9
CH_3_−5’	1.84	*d*	0.9 Hz	14.1
1”				176.7
CH_3_−2”	1.93	*s*		21.9

* …taken from HSQC/HMBC.
